# Analysis of Light Grip Influence on Standing Posture

**DOI:** 10.3390/s21248191

**Published:** 2021-12-08

**Authors:** Angélina Bellicha, Andrés Trujillo-León, Fabien Vérité, Wael Bachta

**Affiliations:** 1 ISIR (Institute of Intelligent Systems and Robotics), UMR 7222 CNRS, Agathe Group INSERM U 1150, Sorbonne Université, 75005 Paris, France; bellicha@isir.upmc.fr (A.B.); verite@isir.upmc.fr (F.V.); 2 Departamento de Electrónica, Universidad de Málaga, 29071 Málaga, Spain; atrujilloleon@uma.es; 3 Instituto de Investigación Biomédica de Málaga (IBIMA), 29071 Málaga, Spain

**Keywords:** postural control, light grip, light touch, healthcare, force sensors

## Abstract

Upright posture control and gait are essential for achieving autonomous daily living activities. Postural control of upright posture relies, among others, on the integration of various sensory information. In this context, light touch (LT) and light grip (LG) of a stationary object provide an additional haptic sensory input that helps to reduce postural sway. When LG was studied through the grasp of a cane, the sensory role of this assistive tool was often limited to a mediation interface. Its role was restricted to transmit the interaction forces between its tip and the ground to the hand. While most studies involve participants standing in an unstable way, such as the tandem stance, in this paper we study LG from a different perspective. We attached a handle of a cane firmly to a stationary support. Thus, we can focus on the role of the hand receptors in the LG mechanism. LG condition was ensured through the tactile information gathered by FSR sensors placed on the handle surface. Moreover, participants involved in our study stood in a usual way. The study involved twelve participants in an experiment composed of two conditions: standing relaxed while lightly gripping an equipped handle attached to the ground, and standing in the same way without gripping the handle. Spatial and frequency analyses confirmed the results reported in the literature with other approaches.

## 1. Introduction

Aging and pathology generally impair postural control [1]. The elderly often use walking canes to avoid falls and their detrimental issues [2]. Post-stroke patients could also benefit from walking canes in order to allow a safer gait and rehabilitation [3,4].

Walking canes provide at least two complementary aids to balance. On the one hand, they offer mechanical support to ensure balance and, on the other hand, they provide sensory information to the central nervous system (CNS). Indeed, the authors of [5,6] showed that light-touching (i.e., with a force below 1 N) a stationary surface with the forefinger halved postural oscillations. Light touch (LT) presumably provides both tactile and proprioceptive information. In fact, the tiny force applied by the finger leads postural oscillations [7], and the arm proprioception influences the effect of LT on sway [8]. The CNS integrates this information with the many other sensory inputs involved in balance to improve its estimation of the upright direction [9].

Since frail people commonly use canes, and light touch improved the mediolateral gait stability of patients with neurological conditions [10], researchers tried to investigate the efficiency of replacing a direct light touch with a light grip (LG) provided by a cane. Jeka et al. investigated, in [11], the influence of LG on balance in healthy participants with eyes shut, and blind participants standing in a tandem position. The tip of the cane was not allowed to move or to slip. The cane handle was lightly gripped by participants but not attached. The study included two cane orientations: perpendicular to the ground and slanted. For each cane orientation, participants carried out two conditions in the experiment. The first condition is an LT contact, while the second is a force contact. In force contact, participants could apply as much force as needed. Participants also performed a control condition without using the cane. The authors reported that touch and force use of the cane reduced the center of pressure (CoP) displacement equivalently compared to the control condition. However, touch contact forces were insufficient to provide mechanical support. The slanted cane orientation decreased sway more than a perpendicular cane.

Albertsen et al., in [12] tried to evaluate the influence of LG on postural sway if the cane tip was allowed to move. Participants, with open eyes, held a cane lightly with three fingers while standing with their feet parallel at a hip-width distance. The experimental work consisted of manipulating the motion of the cane distal tip. Restricting the motion of the tip entirely, or only preventing its motion in the anteroposterior direction, yielded a smaller sway amplitude than standing without any cane. The sway also decreased when letting the tip move freely on the top of a rough surface. In contrast, the authors do not report a significant sway decay if the tip was allowed to move on a slippery surface. The authors argued that the motion of the cane tip on the slippery ground resulted in less transmission of postural oscillations to the fingers.

In [13], the authors studied the effect of light grip provided by a cane in order to compare it to the direct light touch by a fingertip. In other words, they tried to examine the efficiency of using a cane as a mediation of the haptic cues evoked by body oscillations. The participants stood in a tandem position, very unstable in the mediolateral direction. During the study, the authors investigated the effect of two factors: vision and LG. The experiment revealed that lightly gripping a cane reduces postural sway independently of vision.

The above-cited literature considers the cane as a prosthesis. Its sole objective is to transmit the contact forces between the cane tip and the ground to the upper limb. Only the work of [11] used an almost fastened tip, but the cane handle was let free. Moreover, most of the time, the standing position is unstable. In this paper, we would like to investigate the role of the hand in the light grip mechanism. Indeed, the hand possesses many cutaneous receptors and can by itself provide the necessary sensory information. We designed a custom cane handle, equipped it with force sensors, and attached it firmly to the ground. We asked participants to stand in a usual standing way and compared light grip (LG) and no grip (NG) conditions through temporal, spatial, and frequency analyses.

The paper is structured as follows. Firstly, Section 2 describes the experiment performed in this study. This covers the experimental setup, the information about the participants, and the experiment protocol, as well as the data acquisition and processing. Then, the results of the different data analysis are given in Section 3. Finally, in Section 4, the results are discussed and compared to those reported in the literature, and some future questions are addressed.

## 2. Materials and Methods

In this section, the details of the experiment are provided. The experimental setup, characteristics of the participants and the protocol they followed, and the data acquisition and processing are explained.

### 2.1. Participants

Twelve individuals (8 men and 4 women; age =27.3±2.8 years (M±SD),weight = 71.8 ± 15.1 kg, and height =173.4±8.4 cm) participated in the study. Participants were healthy and had no known neurological or muscular disorder. All participants gave informed, written consent as required by the Helsinki declaration (1964) and the local Ethics Committee. All participants were naïve to the goals of the experiment.

### 2.2. Experimental Setup

**Figure 1:**
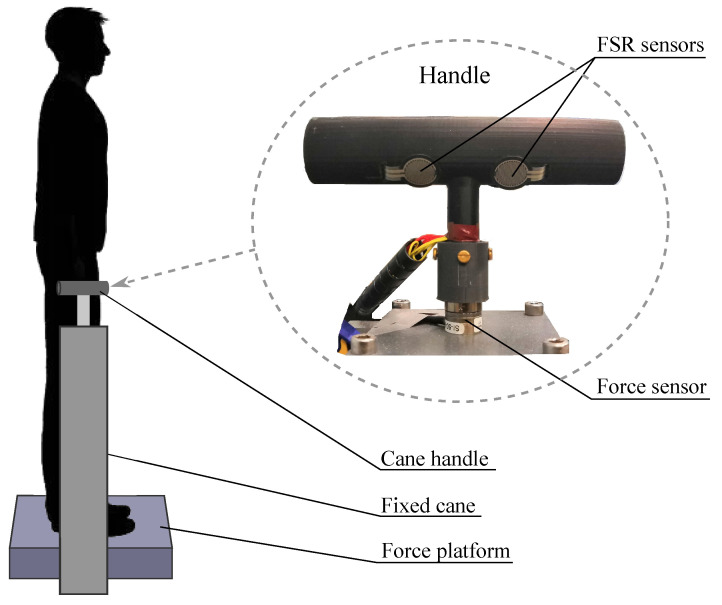
Experimental setup with handle detail.

The experimental setup is shown in Figure 1. It consists of an OR6 (AMTI, Watertown, MA, USA) force platform and a custom, 3D-printed, T-shaped cane handle (see Figure 1 right). The handle was firmly attached to a stable support. The attachment between the handle and the support includes a Nano-17 6-axis force/torque sensor (ATI Industrial Automation, Apex, NC, USA). It recorded the forces and moments applied through the handle–support joint.

**Figure 2:**
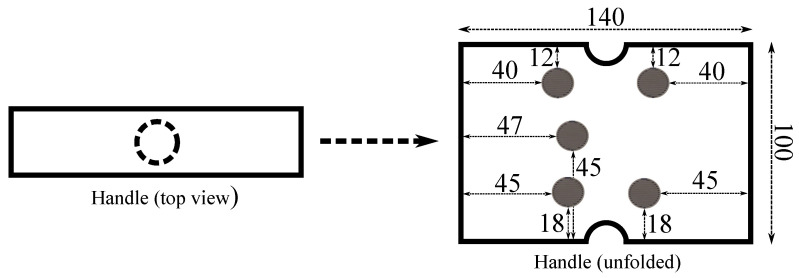
Map of positions of the FSR units on the handle (distances in milimeters).

Keeping the LG condition when grasping the handle was a basic requirement in this paper. For this purpose, force-sensing resistors (FSR) were chosen. This kind of sensors are an economical and effective solution for this task. They were used to capture the gripping force exerted in handles in works such as [14,15] and can be even custom made [16]. In our case, the handle was equipped with five FSR units (FSR402^®^, Interlinks Electronics, Camarillo, CA, USA). They measured the force applied on the handle surface when the user gripped it. They are round-shaped and have an active area with a diameter of 12.7 mm [17]. The location of the sensors can be seen in Figure 2, where an unfolded version of the handle of Figure 1 is shown. The position of each of them was determined after carrying out a preliminary study in which volunteers with different hand sizes and shapes were asked to grasp the handle. The purpose was to cover the most likely gripped surface.

### 2.3. Experimental Procedure

Participants stood upright, barefoot, and relaxed on the top of the OR6 force platform (see Figure 1 left). They had their eyes closed. Their feet were parallel and set apart the width of their hips. The cane handle was placed in an ecological position: it was located beside the force platform and slightly forward. Inappropriate height of the cane usually leads to the worst postural stability and an insecure gait. In a clinic situation, as reported by the authors of [18], cane height can be determined in two ways. It can equal the distance between the ground and either the greater trochanter or the wrist crease. In our study, the experimenter manually adjusted the handle height mid-distance between the two clinical options.

The two experimental conditions were the following:

- Light grip (LG): The participants were lightly gripping the handle with their right hand at the height chosen by the experimenter. Their other arm hung loosely along the body side.
- No grip (NG): The participants had both arms hanging loosely along the body sides.

In order to keep an LG, an audible cue would inform the participants if they applied a force above the fixed threshold so that they could loosen their grip.

Each condition was repeated three times in a randomized order for a total duration under 30 min per participant.

### 2.4. Data Acquisition and Processing

The data acquisition had a double purpose: on the one hand, to ensure the LG condition during the experiment realization and, on the other, to allow the further analysis and computation of the parameters of interest (provided in the sequel).

#### 2.4.1. Light Grip Monitoring Circuit

**Figure 3:**
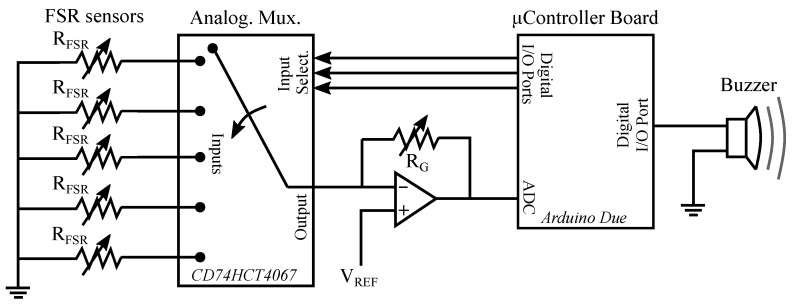
Electronics for the light grip monitoring system.

The electronics that acquired the force measured by the FSR sensors is shown in Figure 3. It is based on an Arduino Due board together with a custom conditioning circuit.

**Figure 4:**
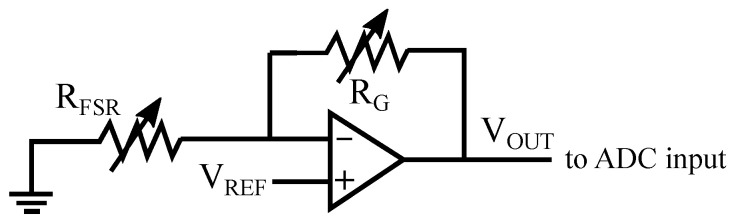
Transimpedance amplifier for the reading of the FSR measure.

As can be seen, one of the terminals of the FSR sensors is grounded. The other is connected to one of the inputs of an analog multiplexer CD74HCT406 (Texas Instruments, Dallas, TX, USA). The selection of the sensor to read is made through the multiplexer control inputs by using the digital ports of the Arduino Due. The chosen FSR unit becomes part of the following circuit (Figure 4):

The circuit is based on an operational amplifier MCP6001 (Microchip Technology Inc., Berkshire, UK) in noninverting configuration. $R_{FSR}$ is the force-dependent resistance on the terminals of the FSR sensor, $V_{REF}$ adjusts the output range, and $R_{G}$ is used to tune the circuit gain. Note that small values of $V_{REF}$ allows having a wider output range. $R_{G}$ value can be used to compensate the choice of a small voltage in $V_{REF}$. The values selected in the experiment were $V_{REF}$ = 0.4 V and $R_{G}$ = 2 KΩ.

**Figure 5:**
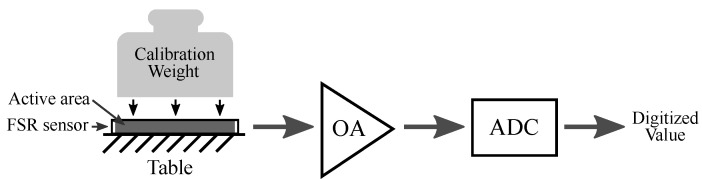
Calibration process: the FSR sensor was fixed to a table surface. Then, a set of calibration weights were placed on the sensor active area. For each weight, the sensor output was conditioned with the operational amplifier stage and digitized with the Arduine Due analog-to-digital converter. The obtained function allows computing the force as $F\left(N\right)=4.348*10^{-3}*Digitized\;Value$, where Digitized Value is in the range [0, 4095].

The output voltage, that will be proportional to the exerted force, is digitized by the analog-to-digital converter of the Arduino Due with a resolution of 12 bits. The FSR sensors are read at a rate of 200 Hz. The system was calibrated in order to obtain the actual force from the FSR sensor read. A set of calibration weights was used for this purpose, as shown in Figure 5.

If any of the sensors detects a force above 1 N, the board microcontroller triggers an alarm by activating a buzzer. As stated in Section 2.3, this audible cue informs the participants that they must loosen their grip, and it only mutes once LG condition is again achieved. The FSR data are not recorded for further analysis.

#### 2.4.2. Postural Data

##### Acquisition

6221 multifunctions cards (National Instruments, Austin, TX, USA) acquired the force platform and the Nano-17 force sensor analog inputs with a resolution of 16 bits. A Simulink real-time computer (Mathworks, Natick, MA, USA) was used to gather and synchronize the data acquisition from both devices at a sampling rate of 2 kHz.

##### Processing

In this paper, we used the CoP position to compute the parameters. This variable was calculated as follows [19]:

(1) $$\begin{array}{c} CoP_{AP}=\frac{-M_{y}+\left(F_{x}*h\right)}{F_{z}} \end{array}$$

(2) $$\begin{array}{c} CoP_{ML}=\frac{M_{x}+\left(F_{y}*h\right)}{F_{z}} \end{array}$$

where $CoP_{AP}$ and $CoP_{ML}$ denote the position of the CoP along the anteroposterior and mediolateral directions. $F_{x}$, $F_{y}$, $F_{z}$, $M_{x}$, and $M_{y}$ are the forces and moments applied by the body on the platform. *h* is the distance between the top of the force plate and its integrated sensors. $CoP_{AP}$, $CoP_{ML}$, and the Nano-17 forces and torques were filtered using a first-order band-pass Butterworth filter with cutting frequencies equal to 0.04 and 2 Hz. The upper band of the band-pass filter is motivated by the upper frequency content of postural sway, which is smaller than two Hertz [20]. The lower band is related to the duration of our experimental trials, equal to 90 s. The 0.04 corresponds approximately to four times the frequency resolution if a fast Fourier transform (FFT) computation had been considered. The first ten seconds of each trial have been removed to discard all the adaptation phenomena that could occur during the beginning of a trial.

The evaluation of postural sway was achieved using the conventional methods reported in the literature [21]:

- *A spatial criterion*: we chose the area of the CoP excursion since it illustrates both the AP and the ML directions. This area was computed as the area of the ellipse encompassing 90% of the CoP points during a trial.
- *A frequency-based criterion*: we computed the power spectral density (PSD) to illustrate the frequency content of the CoP excursion in the AP and ML directions and its dependence on the experimental condition, NG or LG. For each direction (AP or ML), the trials of all participants in each condition (NG or LG) were merged to increase the number of samples. Besides, for both conditions, we used the Matlab function *pwelch* in association with the Hann window. The window length was set to 240 samples and the overlap to 30%. This ensured a good trade-off between the correctness of the result and the smoothing of the PSD. With this tuning, the frequency resolution was about $4.2\;*10^{-3}$ Hz. The total power corresponding to the area under the PSD curves will also be used to discuss the energetics of postural sway.

Matlab function *xcorr* was used to quantify the correlation and the lag between the force exerted on the handle and the evolution of the center of pressure. These parameters were computed separately for the AP and ML directions, i.e., considering the pairs <$f_{AP},CoP_{AP}$> and <$f_{ML},CoP_{ML}$>.

Given the small size of the sample, data is presented as median and interquartile range (IQR). When necessary, nonparametric statistical tests are applied to test the significance of the difference between the NG and LG conditions.

## 3. Results

This section shows the results of the experiment, covering the forces applied on the handle, the analysis of the area of sway, the correlation between the forces applied to the handle and the CoP, and the frequency analysis.

### 3.1. Forces Applied to the Handle

**Figure 6:**
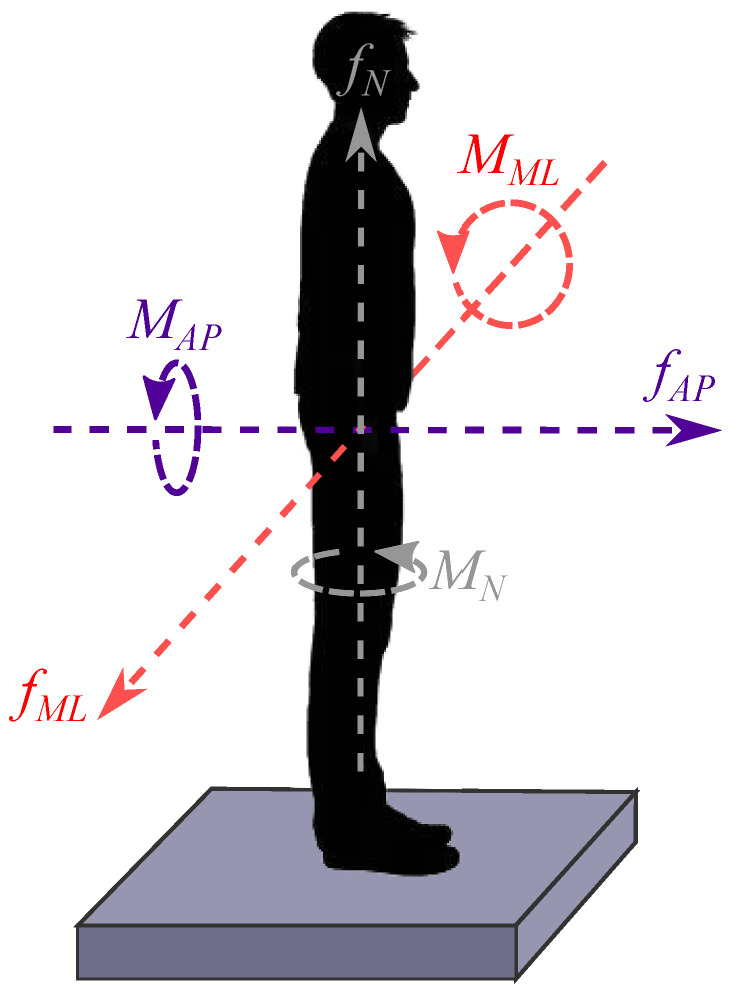
Directions of the forces and moments captured by the handle F/T sensor in the experiment.

**Figure 7:**
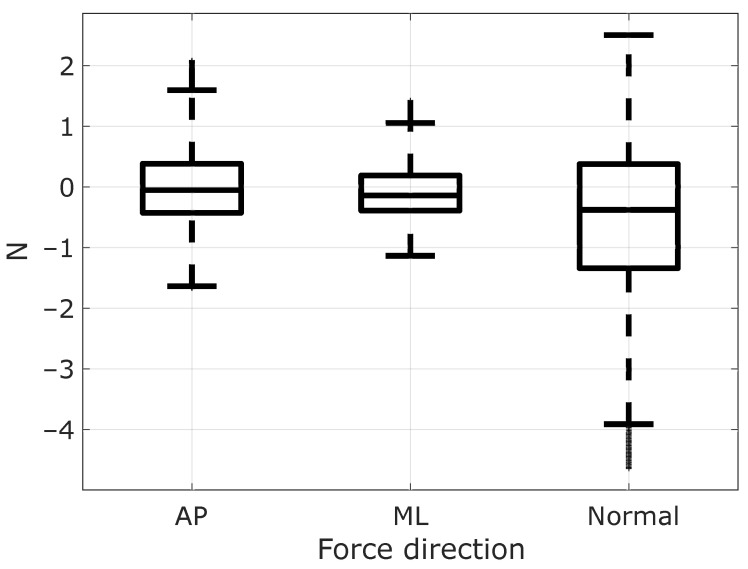
Forces applied to the handle considering the trials of all the participants in the anteroposterior (AP), mediolateral (ML), and normal directions.

**Figure 8:**
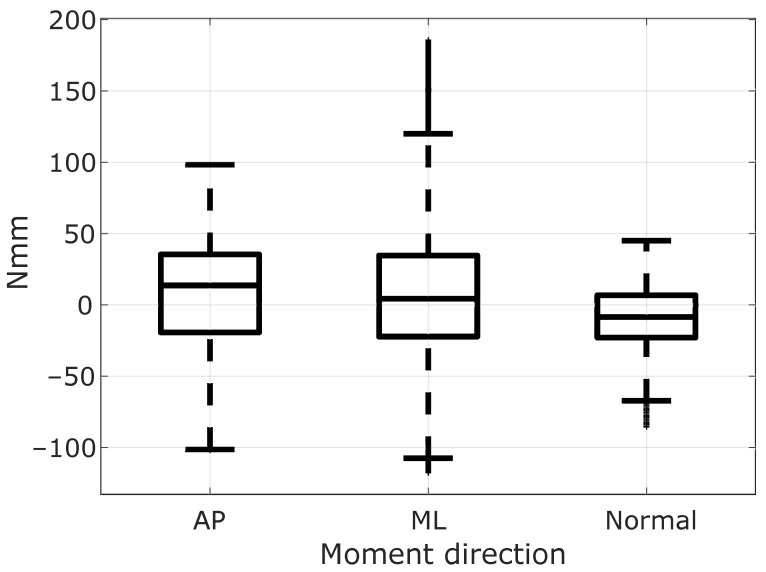
Moments applied to the handle considering the trials of all the participants in the anteroposterior (AP), mediolateral (ML), and normal directions.

The instructions of lightly gripping the handle were observed by participants (Figure 6 helps visualize the directions of the captured forces and moments). Indeed, the recorded data shown in Figure 7 and Figure 8 are low enough to prevent any mechanical assistance. The median and IQR values and forces and torques are given in Table 1.

### 3.2. Reduction of the Area of Sway

**Figure 9:**
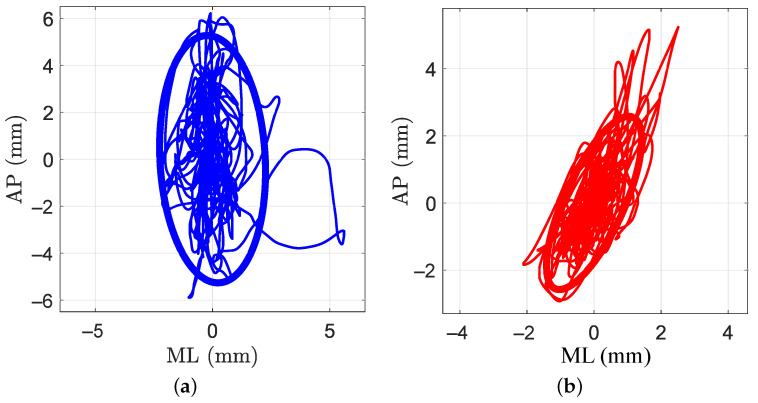
An example of the sway area for a single participant. On the left, the NG condition in blue. On the right, the LG condition in red. (**a**) No grip (NG); (**b**) light grip (LG).

A plot of the area of sway of one participant during one trial in NG and LG conditions is shown in Figure 9. As can be observed, the area in the LG condition is equal to 7.87 mm^2^ and lower than the area computed for the NG condition, which is 37.19 mm^2^.

**Figure 10:**
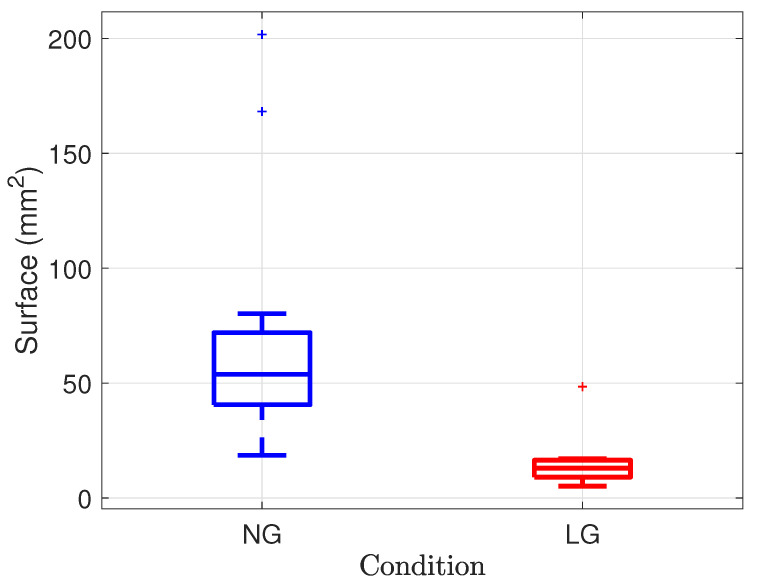
Surface of sway across all the participants trials for the NG (in blue) and LG (in red) conditions.

A Tukey outlier boxplot gathering the surface of sway across all the participants trials is shown in Figure 10; this global result is consistent with that of the participant shown in Figure 9. The area is equal to 53.78 mm^2^ (IQR = 31.38) and 13 mm^2^ (IQR = 7.38) for the NG and LG conditions, respectively. A related samples Wilcoxon signed rank test rejected the medians equality (*p* = 0.002). The LG significantly reduced the sway area.

### 3.3. Correlation between the Forces Applied to the Handle and the Body Sway

**Figure 11:**
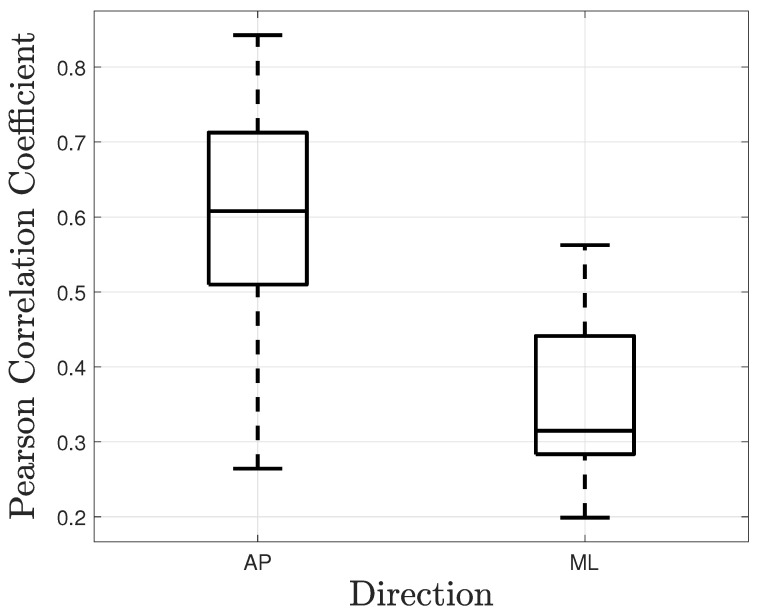
Pearson correlation coefficient between the hand forces applied to the handle and the CoP excursion in the AP and ML directions during the LG condition. All participants were included.

**Figure 12:**
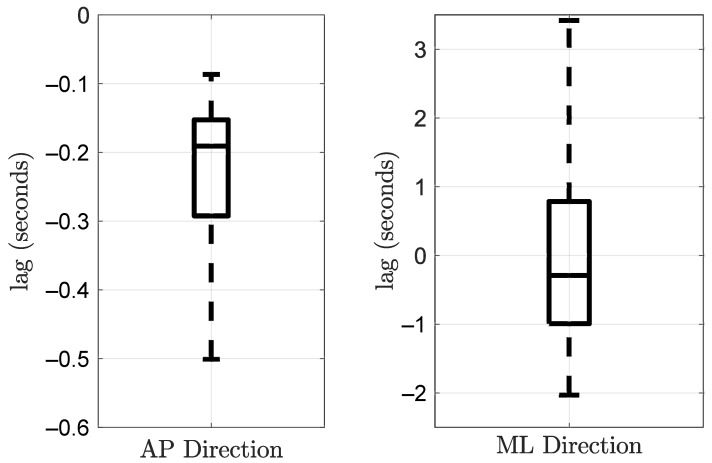
Time lag between the hand forces applied to the handle and the CoP excursion in the AP and ML directions during the LG condition. All participants were included.

**Figure 13:**
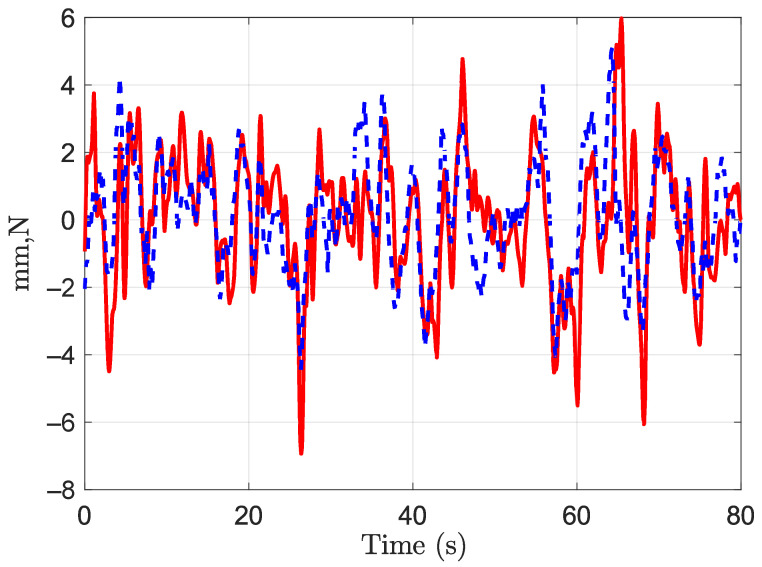
Times series for one participant of the $CoP_{AP}$ in red and 10 $\times \;f_{AP}$ in dashed blue.

**Figure 14:**
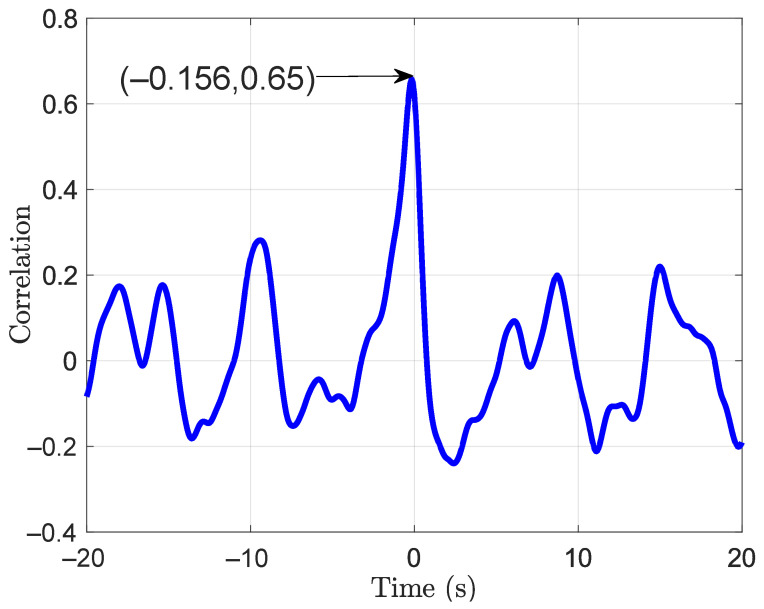
Correlation function for one participant trial between $f_{AP}$ and $CoP_{AP}$.

Pearson correlation and time lag between the force on the handle and the body CoP were calculated. Again, the parameters were computed for the AP and the ML directions separately. Tukey boxplots of the obtained coefficients and time lags are represented in Figure 11 and Figure 12, respectively. The correlation coefficient in the AP direction was $r_{AP}$ = 0.6 (IQR = 0.2). It was greater than the coefficient in the ML direction, $r_{ML}$ = 0.31 (IQR = 0.15). The time lags were −0.191 s (IQR = 0.139) and −0.29 s (IQR = 1.7) in the AP and the ML directions, respectively. Even significantly different from zero, the correlation coefficient in the ML direction is relatively low. The lag in this direction should be taken carefully. A time series and a correlation function in the AP direction are given in Figure 13 and Figure 14, respectively. These plots correspond to a participant trial whose correlation coefficient is near the median. The plots illustrate the similarity between the time series of the two variables.

### 3.4. Frequency Analysis

**Figure 15:**
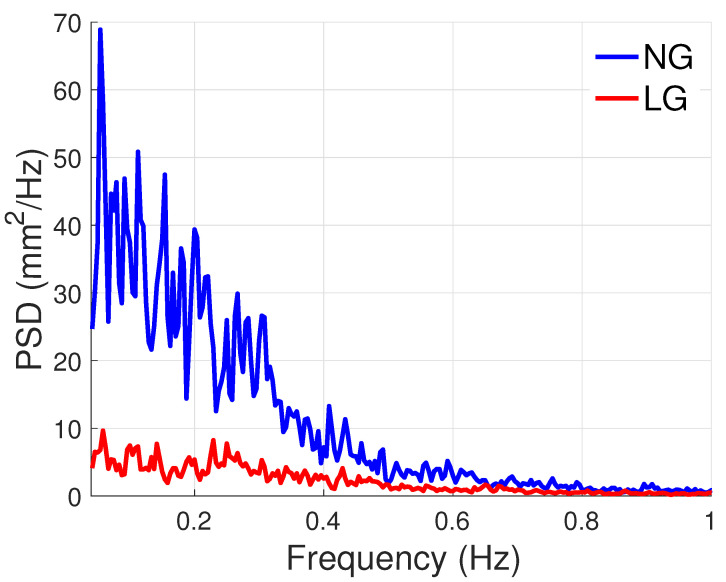
PSD of the $CoP_{AP}$.

**Figure 16:**
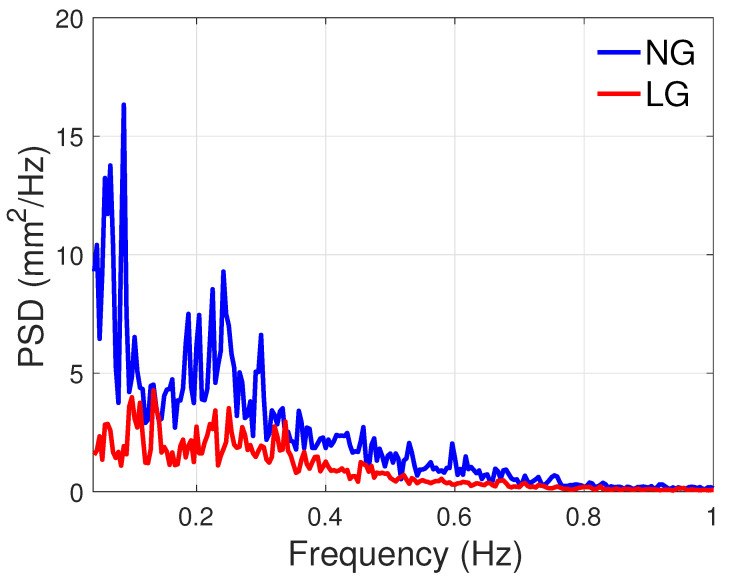
PSD of the $CoP_{ML}$.

PSD plots of the CoP are shown in Figure 15 and Figure 16 for the AP and ML directions. These plots show that power is decreased with LG, especially before 0.4 Hz. The ratio of total power between the NG and LG conditions were 5 and 2.5 in the AP and ML directions, respectively. As can be observed, there is a higher power reduction in the AP direction.

## 4. Discussion and Conclusions

In this work, we designed a setup in which the handle of a cane was firmly attached to a stationary support. This allowed us to focus on the role of the hand receptors in the LG mechanism. The results of our study are in accordance with the literature. In the sequel, these results will be commented and compared to previous studies. Open and future research questions will also be addressed.

### 4.1. Regarding the Light Grip Condition

The FSR sensors ensured a light grip, while the analysis of the forces applied to the cane handle by participants proved insufficient to provide mechanical assistance. Postural instability is more significant in the AP than in the ML direction in the chosen standing position. According to the recorded forces exerted on the handle, the median values of $f_{AP}$ and $M_{ML}$ are equal to −0.05 N and 4.26 Nmm, respectively. Assuming a cane length of 1 m, the resulting torque around the ankle is far less than the approximately 14 Nm reported for normal standing in [22]. The mean of the $f_{Normal}$ in our study is equal to −0.51 N, which is within to the 2 N normal force threshold chosen in [11]. The maximum normal force applied to the handle in our study is 2.5 N, which is bigger than the limit imposed in [13]. However, this force is lower than the 2.7 N which was still considered as a light touch condition in [23].

### 4.2. Light Grip Reduces Sway Area

Light grip reduced postural sway drastically. The median value of the area of sway was divided by four. The percentage of reduction is about 75%, similar to the 50% or more reduction reported in [11]. Our reduction percentage is somehow higher than the one reported by Jeka et al. in [11] and the approximately 20% deduced from the work of Sozzi et al. in [13], since our results cover the whole CoP excursion surface and not a unique direction.

### 4.3. Forces Applied to the Handle Led Postural Sway

The support area is large in the ML direction and narrow in the AP direction when standing with the feet parallel at hip-width, which favors body oscillation in the AP direction [6]. In contrast, the area is narrower in the ML direction during standing in a tandem stance, which favors oscillation in this direction [13].

The standing position of the participants favors postural sway in the AP direction. Thus, the correlation study between the forces on the handle and the CoP displacements was most significant between $f_{AP}$ and $CoP_{AP}$. The Pearson correlation coefficient was higher in the AP direction. Its median value of 0.6 is in line with the approximately 0.5 reported by Jeka et al. in [11]. In contrast, Sozzi et al. reported correlation coefficients ranging from −0.46 to 0.64 when participants were deprived of vision and the tip of the cane was allowed to move freely on the ground. Our results are closer to those reported in [11], and this might be due to the attachment of the distal tip of the cane closer to our experimental design.

The negative values of the obtained median lags, −191 ms and −290 ms in the AP and ML directions, indicate that the small forces applied to the handle led postural sway. They are in accordance with the results reported in [11] and could indicate a long neural loop activated by sensory information. Surprisingly, Sozzi et al. reported in [13] that the cane movements lagged postural oscillations with a very short time, −37.7 (±48.6) ms. This may be due to the lag between the forces applied by the cane tip and its motion.

### 4.4. PSD Analyses Confirm the Sensory Nature of Light Grip

The PSD estimates confirm that the phenomenon in play is sensory. Certainly, the LG condition mainly reduced the frequency components below 0.4 Hz. According to [24], this frequency area corresponds to rambling, i.e., the postural sway component dedicated to the gathering of sensory information.

### 4.5. Concluding Remarks

To sum up, in this paper, we have shown that cane handles firmly attached to the ground provide enough sensory information to reduce postural balance without offering any mechanical support. Even though fingertips are most sensitive to tactile stimulation, the hand palm tactile sensors [25] proved sufficient to gather the necessary information for balance control. However, Alberten et al. in [12] and Sozzi et al. in [13] showed that, under some circumstances, letting the cane end tip move freely reduces postural sway as well. Moreover, Jeka et al. in [11], who studied the haptic influence of a cane with a stationary tip and free handle, showed that the orientation of the cane impacts the sensory-influenced balance reduction.

Both light grips, relying only on the hand sensory receptors and a cane, used as a mediation tool to transfer the cane tip interaction with the ground, appear effective in minimizing sway. Future work could include the investigation of the sensorimotor integration of these two ways of gathering haptic information. A preliminary study would include the design of compact tactile sensing units able to sense tangential forces, as in [26,27,28].

In the midterm, this research will pave the road to the development of affordable technologies, which will alleviate people’s postural problems and offer one solution towards an inclusive society.

## Figures and Tables

**Table 1:** Forces and torques applied to the cane handle.

	*f_AP_*	*f_ML_*	*f_Normal_*	*M_AP_*	*M_ML_*	*M_Normal_*
Median	−0.05 N	−0.14 N	−0.37 N	13.59 Nmm	4.26 Nmm	−8.64 Nmm
IQR	0.8 N	0.57 N	1.7 N	54.68 Nmm	56.91 Nmm	29.59 Nmm

## Data Availability

The data presented in this study are available on request from the corresponding author.

## Abbreviations

The following abbreviations are used in this manuscript:

FSR	Force sensing resistor
CoP	Center of pressure
LT	Light touch
LG	Light grip
NG	No grip
AP	Anteroposterior
ML	Mediolateral
